# Zinc as a phase-specific therapeutic target in hypoglycemia-induced brain injury

**DOI:** 10.1016/j.neurot.2026.e00962

**Published:** 2026-07-09

**Authors:** Dong Gyun Ko, Hyun Wook Yang, Hyun Ho Jeong, Min Kyu Park, Bo Young Choi, Seok Joon Won, Raymond A. Swanson, Sang Won Suh

**Affiliations:** a Department of Neurology, Hallym Neurological Institute, Hallym University Sacred Heart Hospital, Anyang 14068, Republic of Korea; b Department of Physiology, Hallym University, College of Medicine, Chuncheon, Republic of Korea; c Department of Physical Education, Hallym University, Chuncheon, Republic of Korea; d Department of Neurology, University of California, San Francisco, San Francisco, CA, United States of America

**Keywords:** Hypoglycemia, Zinc, Neuron death, Glucose deprivation and reperfusion, Excitotoxicity

## Abstract

Hypoglycemia remains a major cause of neurological morbidity. However, effective targeted therapies for affected brain regions remain lacking. Although excitotoxicity and energy failure have long been implicated, emerging evidence has identified dysregulated zinc signaling as a central mediator of neuronal vulnerability and recovery. During acute glucose deprivation, synapse-released zinc accumulates intracellularly, impairing mitochondrial function, activating nicotinamide adenine dinucleotide phosphate oxidase, amplifying oxidative stress, and triggering poly (ADP-ribose) polymerase-dependent cell death pathways. Notably, neuronal injury is markedly exacerbated during glucose reperfusion, when zinc–reactive oxygen species coupling drives metabolic collapse. During the recovery phase, zinc contributes to neurogenesis, synaptic remodeling, and circuit repair, underscoring its phase-dependent duality. Here, we synthesize mechanistic and translational evidence supporting zinc as a dynamic regulator of neuronal fate in hypoglycemia-induced brain injury. We propose that zinc functions as a metabolic switch linking acute oxidative injury to subsequent regenerative processes. Importantly, this framework suggests a precision-timed therapeutic strategy involving acute zinc chelation or inhibition of zinc-coupled oxidative pathways during injury, followed by controlled restoration of zinc-dependent signaling during recovery. By redefining hypoglycemic brain injury through phase-specific zinc modulation, we identify new therapeutic opportunities relevant not only to hypoglycemia but also to broader metabolic and ischemic brain disorders.

## Introduction

Hypoglycemia, defined as abnormally low blood glucose levels, is a serious metabolic emergency that can precipitate significant neuronal injury and long-term cognitive impairments. The brain relies on a continuous supply of glucose for energy metabolism and is therefore highly vulnerable to even brief periods of glucose deprivation. Although adaptive mechanisms can compensate for transient drops in glucose levels, prolonged or severe hypoglycemia overwhelms these systems, leading to cellular dysfunction and neuronal death. Clinically, hypoglycemia is frequently observed in patients with diabetes mellitus due to insulin therapy or other glucose-lowering agents, though it may occur in critical illness, insulin-secreting tumors, or inborn metabolic disorders [1].

The neurological consequences of hypoglycemia are complex and evolve. Acute episodes may result in seizures, confusion, or coma, whereas recurrent or severe episodes contribute to long-term deficits in memory, learning, and executive function. Neuroimaging studies and neuropathological analyses have consistently demonstrated injury in brain regions such as the hippocampus, cerebral cortex, and basal ganglia, which are rich in excitatory synapses and neurons with high metabolic demands [2].

Historically, research on hypoglycemic brain injury has focused on mechanisms such as adenosine triphosphate (ATP) depletion, ion imbalance, and excitotoxicity mediated by excessive glutamate release [3]. However, emerging evidence has indicated that trace metals, especially zinc, play a critical and previously underappreciated role in hypoglycemia-induced neurodegeneration [4]. Zinc is abundantly stored in presynaptic vesicles of glutamatergic neurons and is co-released with glutamate during periods of heightened neuronal activity or metabolic stress. Under normal physiological conditions, zinc acts as a neuromodulator and antioxidant. However, under pathological conditions such as in hypoglycemia, excessive zinc influx into postsynaptic neurons triggers a cascade of deleterious events, including mitochondrial dysfunction, oxidative stress, and cell death pathway activation [5,6].

Building on this, Suh et al. demonstrated that neuronal injury is not limited to the glucose deprivation phase alone. Paradoxically, neuronal death is often accelerated during the glucose reperfusion period, suggesting that the restoration of glucose itself may exacerbate injury [7]. Zinc appears to be a critical mediator in this biphasic injury pattern, contributing to both initial metabolic disruptions and secondary excitotoxic or oxidative cascades upon glucose reintroduction.

The interplay between zinc signaling, energy metabolism, glutamate excitotoxicity, and neuronal survival under hypoglycemic stress forms a complex and evolving landscape that is still not fully understood. This review aimed to synthesize the current evidence in relation to the role of zinc in hypoglycemia-induced neuronal injury, explore zinc's mechanistic contributions during glucose depletion and reperfusion, and evaluate therapeutic strategies targeting zinc homeostasis. By reframing hypoglycemia not solely as a metabolic insult but also as a zinc-mediated excitotoxic event, we open new avenues for research and therapeutic intervention in metabolic brain injury.

## Hypoglycemia and the Brain

### Pathophysiology, etiology, and prognosis

Hypoglycemia-induced brain injury (neuroglycopenia) results from inadequate glucose availability in the brain. The condition is most commonly triggered by insulin therapy in diabetes, but can also occur in insulinomas, alcohol-related hepatic dysfunction, inborn metabolic disorders, or critical illness. Initial symptoms include tremors, confusion, and palpitations due to sympathetic activation. With prolonged hypoglycemia, neurological symptoms worsen, leading to seizures, coma, or even death. The brain's high metabolic dependence on glucose renders it particularly susceptible to energy failure, especially in neurons with high synaptic activity and limited glycolytic capacity, such as those in the hippocampus and cerebral cortex [8].

Neuronal injury during hypoglycemia typically occurs in phases. The initial insult involves ATP depletion, loss of ion homeostasis, and activation of voltage-gated calcium channels. This initiates a cascade of excitotoxicity, oxidative stress, and cell death [9,10]. Paradoxically, reintroduction of glucose, a necessary step to restore metabolic balance, can exacerbate injury by triggering further reactive oxygen species (ROS) generation and inflammation [7,11]. Neuroimaging and postmortem studies have confirmed preferential vulnerability in the hippocampus, basal ganglia, and cortical layers, areas critical for cognition and memory [12]**.**

### Glucose transport and metabolic adaptation

The brain adapts to mild glucose deficits through enhanced glucose transport across the blood–brain barrier. This is mediated by upregulation of glucose transporters (GLUT1 on endothelial cells and GLUT3 on neurons), driven by hypoglycemia-sensing pathways including adenosine monophosphate-activated protein kinase and hypoxia-inducible factor-1 [8,13]. Astrocytes also play a compensatory role by metabolizing glycogen reserves and providing lactate to neurons [[14], [15], [16]]. However, these adaptations prove inadequate in prolonged or recurrent hypoglycemia.

During energy stress, neurons shift toward anaerobic metabolism, leading to lactate accumulation and tissue acidosis. This metabolic compromise impairs mitochondrial function and ATP production, exacerbating calcium influx and promoting excitotoxicity. Studies in animal models have demonstrated that repeated hypoglycemia impairs glucose transporter expression and reduces metabolic plasticity, thereby increasing the risk of cumulative neuronal injury [17,18].

### Oxidative stress and glutamate excitotoxicity

A key pathological feature of hypoglycemic brain injury is excitotoxicity driven by glutamate accumulation. Under normal conditions, glutamate released into the synaptic cleft is rapidly cleared by astrocytic transporters. However, glucose deprivation impairs this uptake, resulting in excessive extracellular glutamate accumulation. This overstimulates N-methyl-d-aspartate (NMDA) and α-amino-3-hydroxy-5-methyl-4-isoxazolepropionic acid (AMPA) receptors, inducing calcium overload, mitochondrial dysfunction, and ROS generation [9,19].

The loss of ATP during hypoglycemia also disables Na^+^/K^+^-ATPase pumps, leading to membrane depolarization and further glutamate release [12,20]. Additionally, astrocytic dysfunction limits the conversion of glutamate to glutamine, perpetuating excitotoxic cycles. Mitochondria become a major source of superoxide radicals, which react with nitric oxide (NO) to generate peroxynitrite, a highly reactive species that promotes lipid peroxidation and deoxyribonucleic acid (DNA) damage [21] [[22], [23], [24]].

Glutamate-mediated excitotoxicity is closely linked to the downstream activation of cell death pathways, including necrosis, apoptosis, and the unique programmed necrosis known as parthanatos, driven by overactivation of poly (ADP-ribose) polymerase-1 (PARP-1) [25,26]. These cascades also involve additional mediators, including matrix metalloproteinases, inflammatory cytokines, and microglial activation, thereby establishing conditions for secondary injury even after normalization of glucose levels (Table 1).

**Table 1:** Role of zinc in neuronal injury after hypoglycemia.

Mechanism	Key features	Therapeutic implications
Zinc release and translocation	Excessive zinc released from glutamatergic neurons during hypoglycemia accumulates in postsynaptic neurons and mitochondria, disrupting cellular homeostasis	Chelation therapy, modulation of zinc transporters, and neuroprotective agents
Zinc-induced oxidative stress	Zinc activates NADPH oxidase, promotes ROS production and mitochondrial dysfunction; creates a feedback loop with metallothionein-mediated zinc release	Antioxidants, NADPH oxidase inhibitors, and zinc chelators
PARP-1 activation and cell death	Zinc enhances PARP-1 activity, leading to NAD + depletion and AIF-mediated apoptosis (parthanatos); it exacerbates energy failure	PARP-1 inhibitors, zinc modulation, and NAD + precursors
Zinc and microglial activation	Excess zinc triggers microglial activation, increases proinflammatory cytokines, disrupts the BBB, and worsens neuronal injury	Zinc chelators, anti-inflammatory agents, and zinc transporter regulation
Zinc and neurogenesis	Physiological zinc supports stem cell proliferation and BDNF signaling; excess zinc impairs neurogenesis and promotes excitotoxicity	Balanced zinc supplementation or chelation, BDNF-enhancing therapies, transporter modulation

NADPH, nicotinamide adenine dinucleotide phosphate; ROS, reactive oxygen species; PARP-1, poly(ADP-ribose) polymerase-1; NAD^+^, nicotinamide adenine dinucleotide; AIF, apoptosis-inducing factor; BBB, blood–brain barrier; BDNF, brain-derived neurotrophic factor.

## The Role of Zinc in Hypoglycemic Brain Injury

### Zinc translocation and dual role in excitotoxicity

**Fig. 1:**
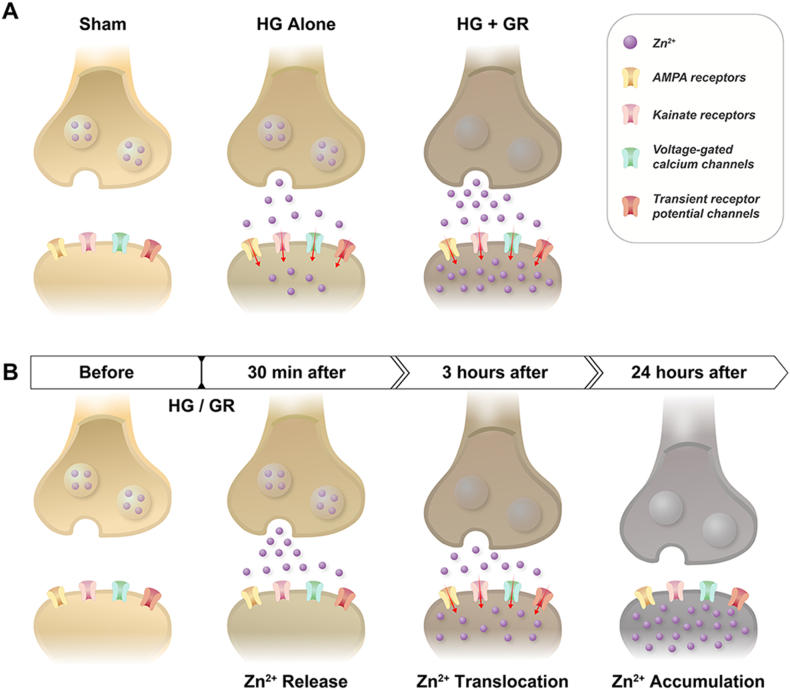
**Distribution of zinc in the synaptic cleft during hypoglycemia.** (A) During severe hypoglycemia, the depletion of glucose leads to excess zinc being released from synaptic vesicles, exacerbating glutamate excitotoxicity in postsynaptic neurons. **(B)** Such cascades initiate within approximately 30 min of hypoglycemia onset. Saturated translocation of zinc from presynaptic to postsynaptic neurons appears 3 h after hypoglycemia. This condition is not reversed following glucose reperfusion; 24 h after hypoglycemia, the full effect of zinc-induced glutamate excitotoxity is observed in postsynaptic neurons, resulting in neuroinflammation. This figure summarizes the temporal dynamics of zinc localization and concentrations during hypoglycemia and subsequent glucose reperfusion.

Zinc is highly concentrated in synaptic vesicles in glutamatergic neurons, particularly in the hippocampus and cortex. Under normal conditions, synaptic zinc modulates neurotransmission by influencing NMDA and AMPA receptor activity. However, during hypoglycemia, zinc is pathologically released into the extracellular space and subsequently taken up by postsynaptic neurons, where it exerts neurotoxic effects [4,12,27] (Fig. 1A).

Intracellular zinc influx occurs via calcium-permeable AMPA/kainate receptors and voltage-gated calcium channels [28,29]. Excess cytosolic zinc disrupts calcium homeostasis, interferes with mitochondrial function, and induces oxidative stress [25,30]. This creates a feedback loop with glutamate excitotoxicity, as the two mechanisms mutually reinforce and amplify each other (Fig. 1B). Zinc also directly inhibits glycolytic enzymes such as glyceraldehyde-3-phosphate dehydrogenase, further reducing ATP production and accelerating neuronal energy failure [31,32]. Fluorescent zinc indicators and knockout models, including zinc transporter 3 (ZnT3)-null mice, confirm that synaptic zinc dysregulation is central to injury progression [33,34]. In mitochondria, zinc triggers permeability transition pore opening and release of pro-apoptotic proteins such as cytochrome *c* [35]. Additionally, zinc also promotes ROS generation by inhibiting respiratory chain complexes, particularly Complex I and III. Zinc-induced mitochondrial swelling and fragmentation precede neuronal death, indicating early mitochondrial compromise [36].

Zinc also activates nicotinamide adenine dinucleotide phosphate (NADPH) oxidase and promotes NO synthase activity, increasing superoxide and peroxynitrite formation [37]. These reactive species damage lipids, proteins, and DNA, as well as stimulate PARP-1, initiating the parthanatos cell death pathway. Zinc-dependent activation of mitogen-activated protein kinase (MAPK) and c-Jun N-terminal kinase signaling cascades also contributes to apoptosis [38]. Interestingly, mitochondria serve both as targets and buffers of zinc. Under stress, mitochondria sequester zinc to delay cytosolic overload, but this buffering capacity is limited and ultimately contributes to mitochondrial failure. Experimental studies using zinc chelators, including N,N,N′,N'-tetrakis (2-pyridylmethyl)ethylenediamine (TPEN), calcium disodium ethylenediaminetetraacetate (Ca-EDTA), demonstrate improved mitochondrial integrity and neuronal survival, emphasizing the therapeutic relevance of zinc modulation [39].

### Glial cell activation and inflammatory response

Zinc dysregulation during hypoglycemia not only affects neurons but also activates glial cells. Microglia respond to hypoglycemic stress with morphological changes, increased proliferation, and secretion of inflammatory mediators such as tumor necrosis factor-α, interleukin (IL)-1β, and IL-6 [40]. Zinc modulates these responses by acting on Toll-like receptors and nuclear factor kappa B pathways, amplifying neuroinflammation [[41], [42], [43]].

Astrocytes, which normally regulate extracellular zinc and glutamate homeostasis, become dysfunctional during hypoglycemia. Zinc impairs astrocytic glutamate uptake by inhibiting excitatory amino acid transporters, increasing extracellular glutamate and excitotoxicity. In addition, zinc accumulation within astrocytes disrupts gap junction communication and mitochondrial function, reducing their ability to support neuronal metabolism [44,45].

The interaction between zinc and glia is bidirectional: activated glial cells release additional zinc, thereby perpetuating a toxic extracellular environment. Furthermore, zinc also induces metallothionein expression, a stress-responsive protein that binds free zinc and modulates its availability. Although initially protective, sustained metallothionein expression may lead to intracellular zinc redistribution and delayed toxicity [46,47].

**Fig. 2:**
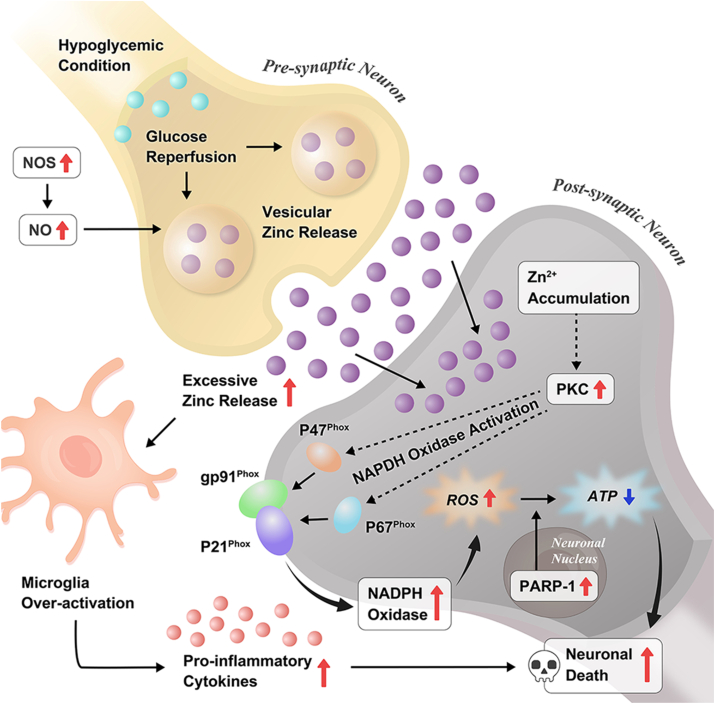
**Role of zinc in hypoglycemic neuronal death.** During severe hypoglycemia, the depletion of glucose impairs neuronal energy metabolism, leading to mitochondrial dysfunction and increased oxidative stress. This metabolic disturbance triggers the pathological release of zinc from presynaptic vesicles and intracellular binding proteins, such as metallothioneins. Excess free zinc accumulates in postsynaptic neurons and mitochondria, where it inhibits respiratory enzymes, promotes reactive oxygen species generation, and disrupts calcium homeostasis. Zinc also activates nicotinamide adenine dinucleotide phosphate oxidase and over-activates poly (ADP-ribose)polymerase-1, resulting in further oxidative damage, nicotinamide adenine dinucleotide depletion, and adenosine triphosphate exhaustion. These cascades culminate in neuronal apoptosis or necrosis. Additionally, zinc promotes microglial activation and the release of proinflammatory cytokines, exacerbating neuronal injury during the glucose reperfusion phase. This figure summarizes the key molecular events by which zinc contributes to neuronal death following hypoglycemia.

Zinc also exerts significant effects on the neurovascular unit. Endothelial cells exposed to hypoglycemia and zinc stress exhibit increased permeability, loss of tight junction proteins, and increased oxidative stress. This compromises blood–brain barrier integrity, allowing infiltration of immune cells and exacerbation of secondary injury [[48], [49], [50]] (Fig. 2). The process can be organized as Table 1.

## Therapeutic Strategies Targeting Zinc in Hypoglycemic Brain Injury

Zinc exhibits dualistic behavior in the brain during hypoglycemia, being neurotoxic in the acute phase and potentially neuroprotective in the recovery phase. Therapeutic interventions must therefore account for these temporal dynamics, aiming to suppress zinc toxicity during acute injury and restore zinc-dependent functions during recovery.

### Acute phase: zinc-limiting therapies and oxidative stress mitigation

#### Zinc chelating agents

Zinc chelation represents the most direct strategy for mitigating acute zinc toxicity. Chelators such as Ca-EDTA and TPEN have demonstrated efficacy in animal models by preventing zinc-induced mitochondrial dysfunction and neuronal death. Intracerebroventricular and systemic administration of these agents reduced infarct volume and improved behavioral outcomes. However, systemic chelation risks disrupting physiological zinc-dependent processes, highlighting the need for central nervous system (CNS)-targeted or transient agents in cases of hypoglycemic encephalopathy [11,39,51,52]. Notably, even delayed administration of zinc chelators following hypoglycemia can confer neuroprotection. In models of global ischemia, which share pathological features with hypoglycemic coma, early or late Ca-EDTA treatment rescued hippocampal CA1 neurons from delayed death, underscoring the pathogenic role of zinc and the therapeutic window for intervention [11]. Small-molecule chelators like clioquinol have exhibited a biphasic effect, where low doses augment, and high doses attenuate oxidative injury, reflecting the complexity of zinc's actions [52]. Overall, these findings support the concept that limiting zinc accumulation during acute hypoglycemia can alleviate neuronal damage.

#### Targeting NMDA receptors

Given the interaction between zinc and glutamatergic signaling, NMDA receptor antagonists offer synergistic protection. Agents such as memantine and ifenprodil reduce calcium influx and excitotoxicity, indirectly attenuating zinc-mediated injury [53,54]. Notably, selective blockade of the NR2B subunit provides neuroprotection without broadly suppressing NMDA function, preserving synaptic plasticity [55,56]. For instance, in ischemic models, selective NR2B antagonism improved outcomes by mitigating both glutamate and zinc toxicity. Clinical translation of such agents remains ongoing, particularly in stroke research, but they hold promise for hypoglycemia-induced injury as well.

#### Managing ROS, NADPH oxidase, and PARP activation

Zinc toxicity amplifies oxidative stress via NADPH oxidase activation and promotes DNA damage, activating PARP-1. Pharmacologically, inhibitors of NADPH oxidase (e.g., apocynin, GKT137831) and PARP (e.g., PJ34, veliparib) preserve nicotinamide adenine dinucleotide levels, prevent energy failure, and inhibit parthanatos [[57], [58], [59]]. Exogenous NO controllers, such as 7-nitroindazole (7-NI), can reduce NO production, thereby limiting peroxynitrite formation and subsequent damage [60,61]. These interventions have demonstrated protective effects in experimental models of hypoglycemia and ischemia, supporting their use as adjunct therapies [62,63]. Hypothermia, a known neuroprotective strategy, may reduce zinc mobilization and ROS generation, offering additive effects with pharmacological agents [64].

#### Antioxidant therapies

Compounds such as N-acetylcysteine (NAC), α-lipoic acid, and vitamin E mitigate zinc-induced ROS damage. NAC replenishes glutathione and enhances metallothionein expression, buffering intracellular zinc [[65], [66], [67], [68]]. Lipoic acid stabilizes mitochondrial membranes and limits zinc-induced lipid peroxidation. Even though preclinical findings are promising, the efficacy of these antioxidants in human hypoglycemia remains under investigation [69].

#### Glial modulation

Controlling microglial and astrocytic activation is crucial for reducing secondary inflammation. Minocycline, a microglial inhibitor, reduces zinc-induced cytokine release and protects against delayed neuronal death [70,71]. Dextromethorphan, an anti-inflammatory agent, and colony-stimulating factor 1 receptor inhibitors (e.g., PLX3397) have also been explored to suppress microglial overactivation. These interventions aim to attenuate the proinflammatory milieu driven by zinc. Additionally, targeting astrocytic glutamate transporters or supporting astrocyte metabolism through monocarboxylate transporter substrates such as lactate may enhance glutamate clearance and limit excitotoxicity. Emerging strategies include boosting astrocyte gap junction communication and antioxidative capacity under zinc stress [72].

## Lactate and Pyruvate Support

**Fig. 3:**
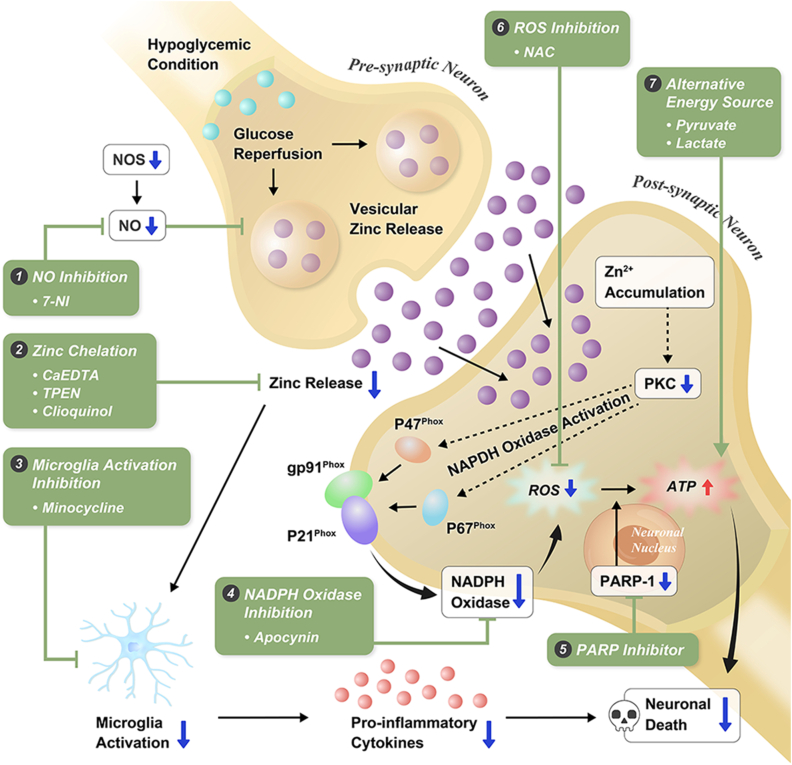
**Possible therapeutic targets for hypoglycemia-induced neuron death.** This schematic illustrates key molecular and cellular mechanisms involved in neuronal death following hypoglycemia, highlighting potential therapeutic intervention points. Hypoglycemia triggers zinc release from synaptic vesicles, leading to its intracellular accumulation and subsequent oxidative stress, mitochondrial dysfunction, and poly (ADP-ribose)polymerase-1 (PARP-1) overactivation. Concurrently, nitric oxide and reactive oxygen species (ROS) further exacerbate zinc-mediated injury. Microglial activation amplifies neuroinflammation, while energy failure impairs neuronal survival. Therapeutic strategies include [1]: zinc chelators to limit zinc accumulation [2]; nicotinamide adenine dinucleotide phosphate oxidase inhibitors to reduce ROS production [3]; PARP-1 inhibitors to preserve nicotinamide adenine dinucleotide and adenosine triphosphate [4]; antioxidants and glutathione precursors to restore redox balance [5]; hypothermia to reduce zinc release and metabolic stress [6]; metabolic substrates such as lactate and pyruvate to support mitochondrial function; and [7] microglial inhibitors to suppress neuroinflammation. Together, these targets form a comprehensive framework for neuroprotection in hypoglycemia-induced brain injury.

Lactate and pyruvate serve as alternative energy substrates for neurons and can help restore mitochondrial function in the context of zinc-induced toxicity. Administration of sodium pyruvate has demonstrated protective effects in ischemic and hypoglycemic models by restoring ATP production and reducing acidosis [73,74]. Lactate shuttling between astrocytes and neurons may help maintain redox balance and support neuronal repair [75,76]. For instance, recurrent hypoglycemia models reveal that exogenous lactate can mitigate oxidative damage and improve cognitive outcomes [12,15,16]. Incorporating metabolic support with substrates that bypass glycolytic blocks offers a complementary approach to zinc-focused therapies (Fig. 3).

### Chronic phase: restoring zinc homeostasis and promoting recovery

#### Zinc deficiency in recovery

Although excess zinc is acutely neurotoxic, zinc deficiency also impairs neurogenesis, synaptic plasticity, and antioxidant defense during recovery. Animal models of chronic hypoglycemia demonstrate downregulation of zinc transporters (e.g., ZnT3, Zrt- and Irt-like protein [ZIP]1), suggesting disrupted zinc signaling [77]. Supplementation with physiologic doses of zinc restores antioxidant enzyme function and synaptic protein expression, aiding in cognitive recovery [78]. For instance, moderate zinc supplementation post-hypoglycemia has been demonstrated to enhance brain-derived neurotrophic factor (BDNF) levels and support hippocampal neurogenesis, which is often blunted following hypoglycemic injury [[79], [80], [81], [82]]. Importantly, dosing is critical; excessive zinc during recovery may be counterproductive; therefore, careful regulation is required.

#### Neurotrophic and neurogenic strategies

**Fig. 4:**
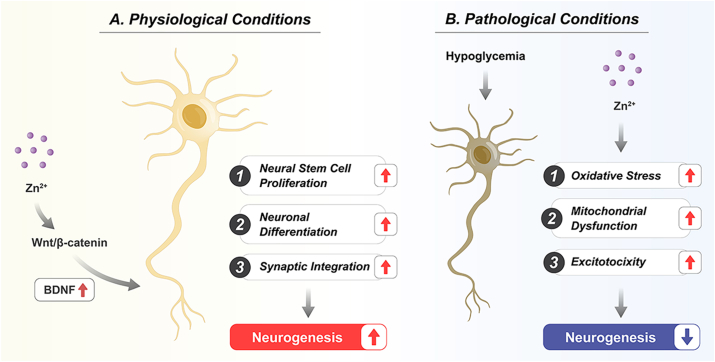
**Zinc's dual role in post-hypoglycemia neurogenesis.** (**A**) Zinc plays a critical yet concentration-dependent role in regulating neurogenesis following hypoglycemia. Under physiological conditions, zinc supports neural stem cell proliferation, neuronal differentiation, and synaptic integration by activating signaling pathways such as Wnt/β-catenin and enhancing brain-derived neurotrophic factor expression. (**B**) During hypoglycemic stress, excess zinc is released from intracellular stores, contributing to oxidative stress, mitochondrial dysfunction, and excitotoxicity. These changes impair the survival and maturation of newly generated neurons in neurogenic regions such as the hippocampus. This figure illustrates this dual role, highlighting how zinc can promote or inhibit neurogenesis depending on intracellular levels and the surrounding redox environment. Restoration of zinc homeostasis may therefore support neuronal regeneration and cognitive recovery following hypoglycemic brain injury.

Encouraging neuronal regeneration is vital following hypoglycemic injury. BDNF and its mimetics (e.g., 7,8-dihydroxyflavone, a TrkB agonist) have been demonstrated to improve cognitive outcomes by promoting neurogenesis and synaptic repair [[83], [84], [85]]. Zinc's role in neurogenesis remains complex. Physiologic zinc is necessary for neural stem cell proliferation and differentiation, whereas excess zinc inhibits these processes [13,86,87] (Fig. 4A and B). Thus, strategies that either supply zinc in deficient states or chelate residual excess can be tailored to individual needs. Enhancing the Wnt/β-catenin pathway and other zinc-sensitive signaling cascades, possibly via pharmacological modulators, offers another avenue to restore neurogenic capacity in the chronic phase.

#### Anti-inflammatory and vascular support

Long-term recovery may be impeded by persistent neuroinflammation and blood–brain barrier damage initiated during hypoglycemia. Chronic use of anti-inflammatory agents, including NSAIDs or specialized pro-resolving mediators, could help resolve inflammation. Additionally, therapies targeting endothelial health, such as exercise, statins, or angiotensin-converting enzyme inhibitors, known to improve endothelial function, might indirectly benefit neuronal recovery by stabilizing the neurovascular unit.

#### Rehabilitation and cognitive training

Finally, cognitive rehabilitation can complement biomedical interventions. Enriched environment and memory training in animal models of hypoglycemia have demonstrated improvements in synaptic plasticity, highlighting that functional recovery is not solely drug-dependent (Table 2).

**Table 2:** Summary of potential therapeutic strategies for hypoglycemia-induced neuronal injury by disease course.

Phase of the disease	Therapeutic target	Mechanism of action	Key agents/Interventions	Challenges
Acute	Zinc chelation	Binds excess zinc to reduce neurotoxicity and oxidative stress	CaEDTA, TPEN, clioquinol, CQ, PBT2	BBB permeability, dose-dependent toxicity
	NMDA receptor antagonists	Block the effect of the NMDA receptor, which is the major route of glutamate surge.	Ketamine, memantine, amantadine	Neuropsychological side effect, too high a dose for neuroprotection
	NO production/NADPH oxidase inhibition	Reduces ROS production, interrupts zinc-ROS amplification loop	7-NI, l-NAME, apocynin, NAC, alpha-lipoic acid, GKT137831, VAS2870	Limited CNS penetration, isoform selectivity, vasodynamic intervention
	Targeted temperature management	Reduce the metabolic rate of cells and the formation of oxidants.	Physical or pharmacological hypothermia to 34∼32 °C	A slower rate of temperature decline is not beneficial in certain clinical populations
	PARP-1 inhibition	Prevents parthanatos and energy collapse	Olaparib, veliparib, PJ34 with glutamate	Risk of impairing DNA repair, BBB permeability, and systemic complications
	Antioxidant therapy	Neutralizes ROS/RNS, supports mitochondrial and zinc buffering systems	Ferulic acid, hyperoside, rutin, and valproic acid	Dose optimization, systemic bioavailability
	Microglia/astrocyte protection	Suppresses proinflammatory cytokine release and NOX2 activation	Minocycline, dextromethorphan, CSF1R inhibitors (e.g., PLX3397)	Avoiding suppression of beneficial microglial functions, the possibility of induced hypoglycemia
	Metabolic support	Sustains ATP production that can be metabolized without NAD+	Lactate, pyruvate	Reperfusion damage
Chronic	Oral zinc intake	Provide zinc for zinc homeostasis and promote the formation of BNDF	Zinc gluconate, ZnCl2	Efficacy problem, dose to not induce additional damage, abnormal neurogenesis
	BDNF/BDNF mimetics	Facilitate TrkB2 receptor and downstream reaction to PI3–K and Ras/MAPK	Recombinant BDNF, 7,8-dihydroxyflavone, LM22-A	BBB penetrance, more preclinical evidence
	Zinc nanoparticle	Increase BBB penetration- both to support or chelate the zinc	PLGA with zinc, ZnO NP	Dose-dependent toxicity

## Discussion

Hypoglycemia-induced brain injury has traditionally been viewed as a metabolic crisis characterized by energy failure, excitotoxic glutamate signaling, and neuronal death. However, accumulating evidence indicates that Zn^2+^ dysregulation is not merely a secondary consequence of metabolic stress but a central mediator that amplifies neuronal injury throughout both the acute and chronic phases of hypoglycemic encephalopathy. The dual role of Zn^2+^ as an essential neuromodulator and a potent neurotoxin under pathological conditions presents both challenges and opportunities for therapeutic intervention.

During acute hypoglycemia, excessive Zn^2+^ is released from presynaptic terminals and accumulates within vulnerable neurons. This pathological redistribution contributes to mitochondrial dysfunction, oxidative stress, calcium dysregulation, PARP-1 activation, and ultimately neuronal death. Moreover, Zn^2+^ also potentiates glutamate excitotoxicity through interactions with glutamate receptors and transport systems, thereby amplifying injury cascades initiated by energy deprivation. Importantly, these mechanisms extend beyond the hypoglycemic period. During glucose reperfusion, renewed metabolic activity generates ROS and further mobilizes intracellular Zn^2+^, producing a secondary wave of injury that resembles ischemia–reperfusion pathology. These observations support a biphasic model of Zn^2+^-mediated neurotoxicity and highlight the importance of therapeutic timing.

Substantial experimental evidence supports Zn^2+^ as a therapeutically actionable target. Zinc chelators, antioxidants, PARP inhibitors, and NMDA receptor antagonists have consistently reduced neuronal degeneration and improved functional outcomes in preclinical models. Nevertheless, global zinc depletion is unlikely to represent a viable clinical strategy, as Zn^2+^ is essential for synaptic transmission, enzymatic activity, gene regulation, and immune function. Consequently, future therapeutic strategies should aim to restore physiological zinc homeostasis rather than indiscriminate zinc depletion.

The role of Zn^2+^ in brain injury extends beyond neuronal death. During the chronic recovery phase, zinc participates in neurogenesis, synaptic remodeling, mitochondrial function, and antioxidant defense. Zinc deficiency following injury may therefore impair endogenous repair mechanisms and contribute to long-term cognitive dysfunction. These observations suggest that the optimal therapeutic approach may involve phase-specific modulation of zinc signaling, with transient suppression of pathological Zn^2+^ accumulation during acute injury followed by preservation or restoration of physiological zinc-dependent processes during recovery.

Increasing evidence also implicates glial cells and the neurovascular unit in Zn^2+^-mediated pathology. Microglial activation, astrocytic dysfunction, blood–brain barrier disruption, and endothelial injury contribute to prolonged neuroinflammation and secondary neurodegeneration. As Zn^2+^ influences metallothionein expression, redox regulation, and inflammatory signaling, zinc dysregulation potentially affects multiple cellular compartments beyond neurons alone. Consequently, therapeutic approaches targeting Zn^2+^ homeostasis may provide broader benefits by simultaneously attenuating neuronal injury, vascular dysfunction, and neuroinflammatory responses.

### Translational perspectives: from Zn^2+^ neurotoxicity mechanisms to therapeutic opportunities

Recent advances in Zn^2+^ biology have identified several mechanistically and clinically relevant therapeutic targets. Among these, modulation of vesicular Zn^2+^ release represents one of the most direct approaches to limiting pathological Zn^2+^ accumulation [88]. ZnT3-dependent vesicular zinc serves as the primary source of activity-dependent synaptic Zn^2+^ release in the forebrain, and experimental studies consistently demonstrate reduced neuronal injury when vesicular zinc signaling is attenuated. Similarly, extracellular zinc chelation has demonstrated neuroprotective effects in experimental models; however, broad zinc depletion remains undesirable due to the essential physiological roles of Zn^2+^.

An alternative strategy involves enhancing endogenous zinc-buffering systems. Metallothioneins represent critical regulators of intracellular zinc homeostasis and oxidative stress responses, and therapeutic enhancement of metallothionein-mediated buffering may simultaneously reduce Zn^2+^ toxicity while preserving physiological zinc signaling. [89,90]. Likewise, selective modulation of ZnT and ZIP transporter families may provide more refined control of pathological zinc redistribution following hypoglycemic injury.

The temporal profile of Zn^2+^ accumulation further suggests that zinc-targeted interventions may be most effective when administered during the early reperfusion period before irreversible mitochondrial dysfunction and oxidative injury become established. Therefore, Zn^2+^-modulating therapies should be considered within the broader framework of reperfusion biology and are likely to function as adjunctive neuroprotective strategies rather than stand-alone treatments. Future clinical applications would most likely involve integration with established reperfusion-based interventions, analogous to current approaches employed in ischemic stroke management.

Several downstream pathways activated by Zn^2+^ overload may also provide clinically accessible therapeutic targets. Mitochondrial dysfunction, oxidative stress, PARP-1 activation, blood–brain barrier disruption, and matrix metalloproteinase activation are all implicated in Zn^2+^-mediated injury. Pharmacological agents targeting these pathways have demonstrated neuroprotective effects in multiple experimental models. Notably, clinically available compounds such as minocycline, N-acetylcysteine, and edaravone may indirectly attenuate Zn^2+^-mediated oxidative and inflammatory injury [[90], [91], [92]], although none were originally developed as zinc-targeted therapies. In addition, PARP inhibitors, including PJ34 and veliparib, have demonstrated promise in preclinical studies, providing broader therapeutic temporal windows than direct zinc chelation [[93], [94], [95]].

A major barrier to clinical translation remains the lack of validated biomarkers capable of assessing Zn^2+^ dysregulation and therapeutic target engagement. Direct visualization of Zn^2+^ dynamics in the human brain remains technically challenging. Emerging approaches, including Zn-sensitive magnetic resonance imaging probes and radionuclide-based imaging using zinc isotopes, offer intriguing possibilities but remain largely experimental [[96], [97], [98], [99], [100]]. Consequently, surrogate biomarkers reflecting zinc homeostasis, including metallothioneins, zinc-regulated proteins, oxidative stress markers, mitochondrial dysfunction, blood–brain barrier injury, and glial activation, may prove useful for patient stratification and therapeutic monitoring. Importantly, the development of validated target-engagement biomarkers remains a key unmet need for future clinical trials [[101], [102], [103], [104], [105], [106]].

The experience with DP-b99 underscores several important lessons for the development of Zn^2+^-targeted therapies. Despite a strong preclinical rationale, the failure of DP-b99 in clinical stroke trials underscores the significance of patient selection, treatment timing, biological target validation, and confirmation of target engagement [[107], [108], [109]]. These lessons suggest that successful clinical translation will require biomarker-guided patient stratification, optimization of therapeutic windows, and integration with contemporary reperfusion-based treatment paradigms, as later studies have confirmed [[110], [111], [112]]. Future Zn^2+^-targeted interventions are therefore most likely to succeed as precision neuroprotective therapies administered to carefully selected patient populations during defined stages of injury progression.

In conclusion, Zn^2+^ is not merely a bystander in hypoglycemia-induced brain injury but a central regulator of neuronal death, neuroinflammation, vascular dysfunction, and recovery. Current evidence supports a conceptual shift from non-selective metal chelation toward precision modulation of Zn^2+^ homeostasis. Continued advances in zinc biology, biomarker development, and translational neuroscience will be essential to determine whether these mechanistic insights can ultimately be translated into clinically meaningful therapies for hypoglycemic encephalopathy and related neurological disorders.

## Author contribution statement

S.W. Suh conceived the study, provided resources to the study and reviewed the manuscript. D.G.Ko investigated the data, developed methodology and drafted the manuscript. H.W. Yang, H·H Jeong, M.K. Park, B.Y. Choi have validated the data. R.A Swanson and S.J. Won reviewed the manuscript. All authors read and approved the final manuscript.

## Funding sources

This study was supported by the 10.13039/501100003725National Research Foundation of Korea (NRF) grant funded by the Korean government (RS-2025-00520396 to Sang Won Suh, RS-2026-25493461 and NRF-2024S1A5C3A01043865 to Bo Young Choi).

## Declaration of competing interest

The authors declare that they have no known competing financial interests or personal relationships that could have appeared to influence the work reported in this paper.

CaEDTA, calcium disodium ethylenediaminetetraacetic acid; TPEN, N,N,N′,N'-tetrakis (2-pyridylmethyl)ethylenediamine; CQ, clioquinol; NMDA, N-methyl-d-aspartate; NO, nitric oxide; NADPH, nicotinamide adenine dinucleotide phosphate; ROS, reactive oxygen species; 7-NI, 7-nitroindazole; l-NAME, Nω-nitro-l-arginine methyl ester; NAC, N-acetylcysteine; CNS, central nervous system; PARP-1, poly (ADP-ribose) polymerase-1; ROS/RNS, reactive oxygen/nitrogen species; NOX2, NADPH oxidase 2; CSF1R, colony-stimulating factor 1 receptor; ATP, adenosine triphosphate; NAD^+^, nicotinamide adenine dinucleotide; BBB, blood–brain barrier; BDNF, brain-derived neurotrophic factor; ZnCl_2_, zinc chloride; TrkB, tropomyosin receptor kinase B; PI3–K, phosphoinositide 3-kinase; MAPK, mitogen-activated protein kinase; PLGA, poly (lactic-*co*-glycolic acid); ZnO NP, zinc oxide nanoparticles.

## References

[1] Cryer P.E. (2007). Hypoglycemia, functional brain failure, and brain death. J Clin Investig.

[2] Auer R.N., Wieloch T., Olsson Y., Siesjö B.K. (1984). The distribution of hypoglycemic brain damage. Acta Neuropathol.

[3] Hu B.R., Kurihara J., Wieloch T. (1995). Persistent translocation and inhibition of Ca2+/calmodulin-dependent protein kinase II in the crude synaptosomal fraction of the vulnerable hippocampus following hypoglycemia. J Neurochem.

[4] Suh S.W., Garnier P., Aoyama K., Chen Y., Swanson R.A. (2004). Zinc release contributes to hypoglycemia-induced neuronal death. Neurobiol Dis.

[5] Koh J.Y., Suh S.W., Gwag B.J., He Y.Y., Hsu C.Y., Choi D.W. (1996). The role of zinc in selective neuronal death after transient global cerebral ischemia. Science.

[6] Tuo Q.Z., Liuyang Z.Y., Lei P., Yan X., Shentu Y.P., Liang J.W. (2018). Zinc induces CDK5 activation and neuronal death through CDK5-Tyr15 phosphorylation in ischemic stroke. Cell Death Dis.

[7] Suh S.W., Gum E.T., Hamby A.M., Chan P.H., Swanson R.A. (2007). Hypoglycemic neuronal death is triggered by glucose reperfusion and activation of neuronal NADPH oxidase. J Clin Investig.

[8] American Diabetes Association Professional Practice C. 6 (2025). Glycemic goals and hypoglycemia: standards of care in Diabetes-2025. Diabetes Care.

[9] Cardoso S., Santos R.X., Correia S.C., Carvalho C., Santos M.S., Baldeiras I. (2013). Insulin-induced recurrent hypoglycemia exacerbates diabetic brain mitochondrial dysfunction and oxidative imbalance. Neurobiol Dis.

[10] Languren G., Montiel T., Ramírez-Lugo L., Balderas I., Sánchez-Chávez G., Sotres-Bayón F. (2019). Recurrent moderate hypoglycemia exacerbates oxidative damage and neuronal death leading to cognitive dysfunction after the hypoglycemic coma. J Cereb Blood Flow Metab.

[11] Calderone A., Jover T., Mashiko T., Noh K.M., Tanaka H., Bennett M.V. (2004). Late calcium EDTA rescues hippocampal CA1 neurons from global ischemia-induced death. J Neurosci.

[12] Auer R.N. (2004). Hypoglycemic brain damage. Metab Brain Dis.

[13] Choi B.Y., Hong D.K., Jeong J.H., Lee B.E., Koh J.Y., Suh S.W. (2020). Zinc transporter 3 modulates cell proliferation and neuronal differentiation in the adult hippocampus. Stem Cell.

[14] Siviy S.M., Walsh J.P., Radisavljevic Z., Cohen R.W., Buchwald N.A., Levine M.S. (1993). Evidence for enhanced synaptic excitation in transplanted neostriatal neurons. Exp Neurol.

[15] Newman L.A., Korol D.L., Gold P.E. (2011). Lactate produced by glycogenolysis in astrocytes regulates memory processing. PLoS One.

[16] Suzuki A., Stern S.A., Bozdagi O., Huntley G.W., Walker R.H., Magistretti P.J. (2011). Astrocyte-neuron lactate transport is required for long-term memory formation. Cell.

[17] Evans M.L., McCrimmon R.J., Flanagan D.E., Keshavarz T., Fan X., McNay E.C. (2004). Hypothalamic ATP-Sensitive K + channels play a key role in sensing hypoglycemia and triggering counterregulatory epinephrine and glucagon responses. Diabetes.

[18] Dong X.X., Wang Y., Qin Z.H. (2009). Molecular mechanisms of excitotoxicity and their relevance to pathogenesis of neurodegenerative diseases. Acta Pharmacol Sin.

[19] L'Amoreaux W.J., Cuttitta C., Santora A., Blaize J.F., Tachjadi J., El Idrissi A. (2010). Taurine regulates insulin release from pancreatic beta cell lines. J Biomed Sci.

[20] Siesjo B.K. (1984). Cell damage in the brain: a speculative synthesis. Acta Psychiatr Scand Suppl.

[21] Swanson R.A., Yu A.C., Chan P.H., Sharp F.R. (1990). Glutamate increases glycogen content and reduces glucose utilization in primary astrocyte culture. J Neurochem.

[22] Brown G.C. (2010). Nitric oxide and neuronal death. Nitric Oxide.

[23] Radi R. (2013). Peroxynitrite, a stealthy biological oxidant. J Biol Chem.

[24] Dienel G.A., Rothman D.L. (2020). Reevaluation of astrocyte-neuron energy metabolism with astrocyte volume fraction correction: impact on cellular glucose oxidation rates, glutamate-glutamine cycle energetics, glycogen levels and utilization rates vs. exercising muscle, and Na(+)/K(+) pumping rates. Neurochem Res.

[25] Brennan-Minnella A.M., Won S.J., Swanson R.A. (2015). NADPH oxidase-2: linking glucose, acidosis, and excitotoxicity in stroke. Antioxid Redox Signal.

[26] Liu L., Li J., Ke Y., Zeng X., Gao J., Ba X. (2022). The key players of parthanatos: opportunities for targeting multiple levels in the therapy of parthanatos-based pathogenesis. Cell Mol Life Sci.

[27] Suh S.W., Hamby A.M., Gum E.T., Shin B.S., Won S.J., Sheline C.T. (2008). Sequential release of nitric oxide, zinc, and superoxide in hypoglycemic neuronal death. J Cereb Blood Flow Metab.

[28] Kalappa B.I., Anderson C.T., Goldberg J.M., Lippard S.J., Tzounopoulos T. (2015). AMPA receptor inhibition by synaptically released zinc. Proc Natl Acad Sci U S A.

[29] Granzotto A., Canzoniero L.M.T., Sensi S.L. (2020). A neurotoxic Menage-a-trois: glutamate, calcium, and zinc in the excitotoxic Cascade. Front Mol Neurosci.

[30] Vermot A., Petit-Härtlein I., Smith S.M.E., Fieschi F. (2021). NADPH oxidases (NOX): an overview from discovery, molecular mechanisms to physiology and pathology. Antioxidants.

[31] Sheline C.T., Behrens M.M., Choi D.W. (2000). Zinc-induced cortical neuronal death: contribution of energy failure attributable to loss of NAD(+) and inhibition of glycolysis. J Neurosci.

[32] Lin W., Mohandas B., Fontaine C.P., Colvin R.A. (2007). Release of intracellular Zn(2+) in cultured neurons after brief exposure to low concentrations of exogenous nitric oxide. Biometals.

[33] Krall R.F., Moutal A., Phillips M.B., Asraf H., Johnson J.W., Khanna R. (2020). Synaptic zinc inhibition of NMDA receptors depends on the association of GluN2A with the zinc transporter ZnT1. Sci Adv.

[34] Krall R., Gale J.R., Ross M.M., Tzounopoulos T., Aizenman E. (2022). Intracellular zinc signaling influences NMDA receptor function by enhancing the interaction of ZnT1 with GluN2A. Neurosci Lett.

[35] Jiang D., Sullivan P.G., Sensi S.L., Steward O., Weiss J.H. (2001). Zn(2+) induces permeability transition pore opening and release of pro-apoptotic peptides from neuronal mitochondria. J Biol Chem.

[36] Pan C.Y., Lin F.Y., Kao L.S., Huang C.C., Liu P.S. (2020). Zinc oxide nanoparticles modulate the gene expression of ZnT1 and ZIP8 to manipulate zinc homeostasis and stress-induced cytotoxicity in human neuroblastoma SH-SY5Y cells. PLoS One.

[37] Islam B.U., Habib S., Ahmad P., Allarakha S., Moinuddin Ali A. (2015). Pathophysiological role of peroxynitrite induced DNA damage in human diseases: a special focus on Poly(ADP-ribose) polymerase (PARP). Indian J Clin Biochem.

[38] Adamo A.M., Zago M.P., Mackenzie G.G., Aimo L., Keen C.L., Keenan A. (2010). The role of zinc in the modulation of neuronal proliferation and apoptosis. Neurotox Res.

[39] Zhao Y., Pan R., Li S., Luo Y., Yan F., Yin J. (2014). Chelating intracellularly accumulated zinc decreased ischemic brain injury through reducing neuronal apoptotic death. Stroke.

[40] Wang Y., Song Y., Zhang L., Huang X. (2024). The paradoxical role of zinc on microglia. J Trace Elem Med Biol.

[41] Kauppinen T.M., Higashi Y., Suh S.W., Escartin C., Nagasawa K., Swanson R.A. (2008). Zinc triggers microglial activation. J Neurosci.

[42] Hongxia L., Yuxiao T., Zhilei S., Yan S., Yicui Q., Jiamin S. (2019). Zinc inhibited LPS-Induced inflammatory responses by upregulating A20 expression in microglia BV2 cells. J Affect Disord.

[43] Feijo G.D.S., Jantsch J., Correia L.L., Eller S., Furtado-Filho O.V., Giovenardi M. (2022). Neuroinflammatory responses following zinc or branched-chain amino acids supplementation in Obese rats. Metab Brain Dis.

[44] Alirezaei M., Mordelet E., Rouach N., Nairn A.C., Glowinski J., Prémont J. (2002). Zinc-induced inhibition of protein synthesis and reduction of connexin-43 expression and intercellular communication in mouse cortical astrocytes. Eur J Neurosci.

[45] Suh S.W., Aoyama K., Alano C.C., Anderson C.M., Hamby A.M., Swanson R.A. (2007). Zinc inhibits astrocyte glutamate uptake by activation of poly(ADP-ribose) polymerase-1. Mol Med.

[46] Hong D.K., Kho A.R., Lee S.H., Jeong J.H., Kang B.S., Kang D.H. (2020). Transient receptor potential melastatin 2 (TRPM2) inhibition by antioxidant, N-Acetyl-l-Cysteine, reduces global cerebral ischemia-induced neuronal death. Int J Mol Sci.

[47] Ruan Z., Zhang D., Huang R., Sun W., Hou L., Zhao J. (2022). Microglial activation damages dopaminergic neurons through MMP-2/-9-Mediated increase of blood-brain barrier permeability in a parkinson's disease mouse model. Int J Mol Sci.

[48] Qi Z., Liang J., Pan R., Dong W., Shen J., Yang Y. (2016). Zinc contributes to acute cerebral ischemia-induced blood-brain barrier disruption. Neurobiol Dis.

[49] Qin W., Li J., Zhu R., Gao S., Fan J., Xia M. (2019). Melatonin protects blood-brain barrier integrity and permeability by inhibiting matrix metalloproteinase-9 via the NOTCH3/NF-kappaB pathway. Aging (Albany NY).

[50] Yang X., Li W., Ding M., Liu K.J., Qi Z., Zhao Y. (2024). Contribution of zinc accumulation to ischemic brain injury and its mechanisms about oxidative stress, inflammation, and autophagy: an update. Metallomics.

[51] Frederickson C.J., Suh S.W., Koh J.Y., Cha Y.K., Thompson R.B., LaBuda C.J. (2002). Depletion of intracellular zinc from neurons by use of an extracellular chelator in vivo and in vitro. J Histochem Cytochem.

[52] Oyama T.M., Oyama K., Fukunaga E., Ishibashi H., Oyama Y. (2014). Clioquinol, a lipophilic Zn2+ chelator, augments and attenuates the cytotoxicity of H2O2: a bell-shaped response curve of the effects of the drug. Toxicol Res.

[53] Kutzing M.K., Luo V., Firestein B.L. (2012). Protection from glutamate-induced excitotoxicity by memantine. Ann Biomed Eng.

[54] Hohmann U., Ghadban C., Hohmann T., Kleine J., Schmidt M., Scheller C. (2022). Nimodipine exerts time-dependent neuroprotective effect after excitotoxical damage in organotypic slice cultures. Int J Mol Sci.

[55] Picconi B., Tortiglione A., Barone I., Centonze D., Gardoni F., Gubellini P. (2006). NR2B subunit exerts a critical role in postischemic synaptic plasticity. Stroke.

[56] Liu Y., Wong T.P., Aarts M., Rooyakkers A., Liu L., Lai T.W. (2007). NMDA receptor subunits have differential roles in mediating excitotoxic neuronal death both in vitro and in vivo. J Neurosci.

[57] Mazzone G.L., Nistri A. (2011). Effect of the PARP-1 inhibitor PJ 34 on excitotoxic damage evoked by kainate on rat spinal cord organotypic slices. Cell Mol Neurobiol.

[58] Puentes L.N., Lengyel-Zhand Z., Reilly S.W., Mach R.H. (2021). Evaluation of a low-toxicity PARP inhibitor as a neuroprotective agent for parkinson's disease. Mol Neurobiol.

[59] Yang T., Zang D.-W., Shan W., Guo A.-C., Wu J.-P., Wang Y.-J. (2019). Synthesis and evaluations of novel apocynin derivatives as anti-glioma agents. Front Pharmacol.

[60] Vitcheva V., Simeonova R., Kondeva-Burdina M., Mitcheva M. (2015). Selective nitric oxide synthase inhibitor 7-Nitroindazole protects against cocaine-induced oxidative stress in rat brain. Oxid Med Cell Longev.

[61] Favié L.M.A., Cox A.R., van den Hoogen A., Nijboer C.H.A., Peeters-Scholte C.M.P.C.D., van Bel F. (2018). Nitric oxide synthase inhibition as a neuroprotective strategy following hypoxic–ischemic encephalopathy: evidence from animal studies. Front Neurol.

[62] Sun Y.J., Zhang Z.Y., Fan B., Li G.Y. (2019). Neuroprotection by therapeutic hypothermia. Front Neurosci.

[63] Hong D.K., Park Y.S., Woo J.S., Kim J.H., Beom J.H., Chung S.P. (2021). Transient global ischemia-induced brain inflammatory cascades attenuated by targeted temperature management. Int J Mol Sci.

[64] Shin B.S., Won S.J., Yoo B.H., Kauppinen T.M., Suh S.W. (2010). Prevention of hypoglycemia-induced neuronal death by hypothermia. J Cereb Blood Flow Metab.

[65] Aoyama K., Watabe M., Nakaki T. (2008). Regulation of neuronal glutathione synthesis. J Pharmacol Sci.

[66] Ferner R.E., Dear J.W., Bateman D.N. (2011). Management of paracetamol poisoning. Bmj.

[67] Ezerina D., Takano Y., Hanaoka K., Urano Y., Dick T.P. (2018). N-Acetyl cysteine functions as a fast-acting antioxidant by triggering intracellular H(2)S and sulfane sulfur production. Cell Chem Biol.

[68] Lee M., Ko D.G., Hong D.K., Lim M.S., Choi B.Y., Suh S.W. (2020). Role of excitatory amino acid carrier 1 (EAAC1) in neuronal death and neurogenesis after ischemic stroke. Int J Mol Sci.

[69] Jibril A.T., Jayedi A., Shab-Bidar S. (2022). Efficacy and safety of oral alpha-lipoic acid supplementation for type 2 diabetes management: a systematic review and dose-response meta-analysis of randomized trials. Endocr Connect.

[70] Kobayashi K., Imagama S., Ohgomori T., Hirano K., Uchimura K., Sakamoto K. (2013). Minocycline selectively inhibits M1 polarization of microglia. Cell Death Dis.

[71] Lu Q., Xiong J., Yuan Y., Ruan Z., Zhang Y., Chai B. (2022). Minocycline improves the functional recovery after traumatic brain injury via inhibition of aquaporin-4. Int J Biol Sci.

[72] Zhang B., Ran Y., Wu S., Zhang F., Huang H., Zhu C. (2021). Inhibition of colony stimulating factor 1 receptor suppresses neuroinflammation and neonatal hypoxic-ischemic brain injury. Front Neurol.

[73] Suh S.W., Aoyama K., Matsumori Y., Liu J., Swanson R.A. (2005). Pyruvate administered after severe hypoglycemia reduces neuronal death and cognitive impairment. Diabetes.

[74] Kovalenko T.N., Ushakova G.A., Osadchenko I., Skibo G.G., Pierzynowski S.G. (2011). The neuroprotective effect of 2-oxoglutarate in the experimental ischemia of hippocampus. J Physiol Pharmacol.

[75] Goodwin M.L., Gladden L.B., Nijsten M.W.N. (2020). Lactate-protected hypoglycemia (LPH). Front Neurosci.

[76] Su G., Farhat R., Laxman A.K., Chapman-Natewa K., Nelson I.E., Chan O. (2023). Astrocyte glycogen is a major source of hypothalamic lactate in rats with recurrent hypoglycemia. Diabetes.

[77] Suh S.W., Won S.J., Hamby A.M., Yoo B.H., Fan Y., Sheline C.T. (2009). Decreased brain zinc availability reduces hippocampal neurogenesis in mice and rats. J Cereb Blood Flow Metab.

[78] Wessels I., Maywald M., Rink L. (2017). Zinc as a gatekeeper of immune function. Nutrients.

[79] Nowak G., Legutko B., Szewczyk B., Papp M., Sanak M., Pilc A. (2004). Zinc treatment induces cortical brain-derived neurotrophic factor gene expression. Eur J Pharmacol.

[80] Hwang I.Y., Sun E.S., An J.H., Im H., Lee S.H., Lee J.Y. (2011). Zinc-triggered induction of tissue plasminogen activator by brain-derived neurotrophic factor and metalloproteinases. J Neurochem.

[81] Szewczyk B. (2013). Zinc homeostasis and neurodegenerative disorders. Front Aging Neurosci.

[82] Choi B.Y., Kim I.Y., Kim J.H., Lee B.E., Lee S.H., Kho A.R. (2017). Administration of zinc plus Cyclo-(His-Pro) increases hippocampal neurogenesis in rats during the early phase of streptozotocin-induced diabetes. Int J Mol Sci.

[83] Kempermann G., Song H., Gage F.H. (2015). Neurogenesis in the adult hippocampus. Cold Spring Harb Perspect Biol.

[84] Chen C., Wang Z., Zhang Z., Liu X., Kang S.S., Zhang Y. (2018). The prodrug of 7,8-dihydroxyflavone development and therapeutic efficacy for treating alzheimer's disease. Proc Natl Acad Sci USA.

[85] Wang C.S., Kavalali E.T., Monteggia L.M. (2022). BDNF signaling in context: from synaptic regulation to psychiatric disorders. Cell.

[86] Levenson C.W., Morris D. (2011). Zinc and neurogenesis: making new neurons from development to adulthood. Adv Nutr.

[87] Choi B.Y., Kim I.Y., Kim J.H., Lee B.E., Lee S.H., Kho A.R. (2016). Zinc plus cyclo-(his-pro) promotes hippocampal neurogenesis in rats. Neuroscience.

[88] Medvedeva Y.V., Lin B., Shuttleworth C.W., Weiss J.H. (2009). Intracellular Zn^2+^ accumulation contributes to synaptic failure, mitochondrial depolarization, and cell death in an acute slice oxygen–glucose deprivation model of ischemia. J Neurosci.

[89] Martinez de Lizarrondo S., Gakuba C., Herbig B.A., Repessé Y., Ali C., Denis C.V. (2017). Potent thrombolytic effect of N-acetylcysteine on arterial thrombi. Circulation.

[90] Vivien D., Lebatard S., Mazighi M., Brikci-Nigass N., Desilles J.P., Tomadesso C. (2025). N-acetylcysteine (NAC) as an adjunct to intravenous fibrinolysis in patients with acute ischemic stroke: a single group study (NAC-Safety). Neuroscience.

[91] Zhao K., Wang P., Tang X., Chang N., Shi H., Guo L. (2023). The mechanisms of minocycline in alleviating ischemic stroke damage and cerebral ischemia-reperfusion injury. Eur J Pharmacol.

[92] Xiao P., Huang H., Zhao H., Liu R., Sun Z., Liu Y. (2024). Edaravone dexborneol protects against cerebral ischemia/reperfusion-induced blood-brain barrier damage by inhibiting ferroptosis via activation of nrf-2/HO-1/GPX4 signaling. Free Radic Biol Med.

[93] Chen J., Li X., Xu S., Zhang M., Wu Z., Zhang X. (2020). Delayed PARP-1 inhibition alleviates post-stroke inflammation in Male versus female mice: differences and similarities. Front Cell Neurosci.

[94] Haddad M., Beray-Berthat V., Coqueran B., Plotkine M., Marchand-Leroux C., Margaill I. (2013). Combined therapy with PJ34, a poly(ADP-ribose)polymerase inhibitor, reduces tissue plasminogen activator-induced hemorrhagic transformations in cerebral ischemia in mice. Fundam Clin Pharmacol.

[95] Koehler R.C., Bedirian K., Chen M.H., Shi Y., Cao S., Avery B.D. (2025). Multicenter stroke preclinical assessment network analysis of cardiovascular risk factor subgroups treated with the Poly(ADP-Ribose) polymerase inhibitor veliparib. J Am Heart Assoc.

[96] Chen L., Shen Q., Liu Y., Zhang Y., Sun L., Ma X. (2025). Homeostasis and metabolism of iron and other metal ions in neurodegenerative diseases. Signal Transduct Targeted Ther.

[97] Clavijo Jordan M.V., Lo S.T., Chen S., Preihs C., Chirayil S., Zhang S. (2016). Zinc-sensitive MRI contrast agent detects differential release of Zn(II) ions from the healthy vs. malignant mouse prostate. Proc Natl Acad Sci USA.

[98] Major J.L., Parigi G., Luchinat C., Meade T.J. (2007). The synthesis and *In Vitro* testing of a zinc-activated MRI contrast agent. Proc Natl Acad Sci.

[99] Firth G., Yu Z., Bartnicka J.J., Parker D., Kim J., Sunassee K. (2022). Imaging zinc trafficking in vivo by positron emission tomography with zinc-62. Metallomics.

[100] Mu C., Liu X., Ali U., Larson P.E.Z., Ho S.P., Flavell R.R. (2026). Molecular imaging of in vivo zinc distribution using nuclear and magnetic resonance imaging. Chem Biomed Imag.

[101] Holper L., Lan M.J., Brown P.J., Sublette E.M., Burke A., Mann J.J. (2019). Brain cytochrome-c-oxidase as a marker of mitochondrial function: a pilot study in major depression using NIRS. Depress Anxiety.

[102] Candelario-Jalil E., Thompson J., Taheri S., Grossetete M., Adair J.C., Edmonds E. (2011). Matrix metalloproteinases are associated with increased blood-brain barrier opening in vascular cognitive impairment. Stroke.

[103] Li X., Wang X., Yang Y., Zhou J., Wu X., Zhao J. (2024). Elevated plasma matrix metalloproteinase 9 in schizophrenia patients associated with poor antipsychotic treatment response and white matter density deficits. Schizophrenia.

[104] Park K.P., Rosell A., Foerch C., Xing C., Kim W.J., Lee S. (2009). Plasma and brain matrix metalloproteinase-9 after acute focal cerebral ischemia in rats. Stroke.

[105] Lee J.S., Yoon B.S., Kim Y., Park C.B. (2024). LDHB-Deficient brain exhibits resistance to ischemic neuronal cell death due to increased vasodilation. Biochem Biophys Res Commun.

[106] Dong F., Wang X., Li J., Zhao D., Li J. (2024). Causal relationship between lactate dehydrogenase and risk of developing ischemic stroke: a Mendelian randomized study. Brain Behav.

[107] Rosenberg G., Angel I., Kozak A. (2005). Clinical pharmacology of DP-b99 in healthy volunteers: first administration to humans. Br J Clin Pharmacol.

[108] Diener H.-C., Schneider D., Lampl Y., Bornstein N.M., Kozak A., Rosenberg G. (2008). DP-b99, a membrane-activated metal ion chelator, as neuroprotective therapy in ischemic stroke. Stroke.

[109] Lees K.R., Bornstein N., Diener H.-C., Gorelick P.B., Rosenberg G., Shuaib A. (2013). Results of membrane-activated chelator stroke intervention randomized trial of DP-b99 in acute ischemic stroke. Stroke.

[110] Lang W., Stadler C.H., Poljakovic Z., Fleet D. (2013). A prospective, randomized, placebo-controlled, double-blind trial about safety and efficacy of combined treatment with alteplase (rt-PA) and cerebrolysin in acute ischaemic hemispheric stroke. Int J Stroke.

[111] Khasanova D.R., Kalinin M.N. (2023). Cerebrolysin as an early Add-on to reperfusion therapy: risk of hemorrhagic transformation after ischemic stroke (CEREHETIS), a prospective, randomized, multicenter pilot study. BMC Neurol.

[112] Muresanu D.F., Heiss W.D., Hoemberg V., Bajenaru O., Popescu C.D., Vester J.C. (2016). Cerebrolysin and recovery after stroke (CARS): a randomized, placebo-controlled, double-blind, multicenter trial. Stroke.

